# Role of high-field intraoperative magnetic resonance imaging on a multi-image fusion-guided stereotactic biopsy of the basal ganglia: A case report

**DOI:** 10.3892/ol.2014.2680

**Published:** 2014-11-07

**Authors:** XIANG SUN, ZHIJUAN CHEN, SHUYUAN YANG, JIANNING ZHANG, SHUYUAN YUE, ZENGGUANG WANG, WEIDONG YANG

**Affiliations:** Department of Neurosurgery, Tianjin Medical University General Hospital, Tianjin 300052, P.R. China

**Keywords:** intraoperative magnetic resonance imaging, multi-image fusion, stereotactic, biopsy

## Abstract

The aim of the present case study was to investigate the advantages of intraoperative magnetic resonance imaging (iMRI) on the real-time guidance and monitoring of a stereotactic biopsy. The study describes a patient with intracranial lesions, which were examined by conventional MRI and diffusion tensor imaging using a 1.5T intraoperative MRI system. The digital and pre-operative positron emission/computed tomography image data were transferred to a BrainLAB planning workstation, and a variety of images were automatically fused. The BrainLAB software was then used to reconstruct the corticospinal tract (CST) and create a three-dimensional display of the anatomical association between the CST and the brain lesions. A Leksell surgical planning workstation was used to identify the ideal target site and a reasonable needle track for the biopsy. The 1.5T iMRI was used to effectively monitor the intracranial condition during the brain biopsy procedure. Post-operatively, the original symptoms of the patient were not aggravated and no further neurological deficits were apparent. The histopathological diagnosis of non-Hodgkin’s B-cell lymphoma was made. Using high-field iMRI, the multi-image fusion-guided stereotactic brain biopsy allows for a higher positive rate of biopsy and a lower incidence of complications. The approach of combining multi-image fusion images with the frame-based stereotactic biopsy may be clinically useful for intracranial lesions of deep functional areas.

## Introduction

Intraoperative magnetic resonance imaging (iMRI) has the advantage of real-time guidance and monitoring of the surgical procedure. Furthermore, high-field iMRI can generate high-quality anatomical and functional images. In recent years, with the use of iMRI, frameless stereotactic brain biopsies have been introduced into the clinic (1–3). Frame-based stereotactic brain biopsies remain the gold-standard procedure for achieving accuracy, a shorter surgical time, fewer complications and a higher positive rate of biopsy (4,5). To the best of our knowledge, the effects of high-field iMRI in patients with basal ganglia region lesions who have undergone a frame-based stereotactic brain biopsy have not yet been discussed in the literature. Furthermore, no study with this design, combining MRI-positron emission tomography (PET)-diffusion tensor imaging (DTI) fusion imaging with the stereotactic biopsy of a basal ganglia lesion, has been performed until now. In the present study, a deep lesion located in the area of the left internal capsule is described. A needle biopsy was successfully performed and a pathological diagnosis was explicitly obtained. Written informed consent was obtained from the patient.

## Case report

A 68-year-old right-handed female presented with slurred speech and a weakness of the right extremities five days prior to admission to the Tianjin Medical University General Hospital (Tianjin, China). These symptoms were not accompanied by a headache, vomiting or fever. In addition, the patient’s medical history contained no previous condition that could have contributed to the symptoms. On the first day of hospitalization, tests revealed a body temperature of 36.4°C (normal range, 36–37°C), an oxygen saturation level of 97% (normal range, ≤94°C), a blood pressure of 135/85 mmHg (normal range, 90–140/60–90 mmHg), a pulse rate of 78 beats/min (normal range, 60–100 beats/min) and a respiratory rate of 19 breaths/min (normal range, 12–20 breaths/min). Neurological examinations revealed slurred speech, weakness of the right extremities (as demonstrated by a grade IV muscle power score, measured by the manual muscle test) and the presence of positive pathological reflexes of the right lower extremity. An immediate MRI head scan identified left basal ganglia lesions with a slightly long signal upon T1-weighted imaging (T1WI) and a long signal upon T2WI. Subsequent to a gadolinium-diethylenetriaminepentaacetic acid-enhanced scan, the lesions appeared abnormal. The lumbar puncture revealed a normal cerebrospinal fluid (CSF) pressure and normal outward appearance during an unremarkable test. However, the results of the CSF biochemical contents revealed levels of 1.5 g/l protein (normal range, 0.15–0.45 g/l), 0.5 mmol/l glucose (normal range, 2.5–4.4 mmol/l) and 144 mmol/l sodium chloride (normal range, 120–130 mmol/l). With the exception of the lumbar puncture, the laboratory examinations identified no notable results. An ^18^F-fluorodeoxyglucose (FDG)-PET scan of the brain, performed two days prior to the surgery with the Discovery LS PET/computed tomography (CT) scanner (GE Medical Systems, Waukesha, WI, USA), revealed a focus of increased FDG uptake in the area of the left basal ganglia.

**Figure 1:**
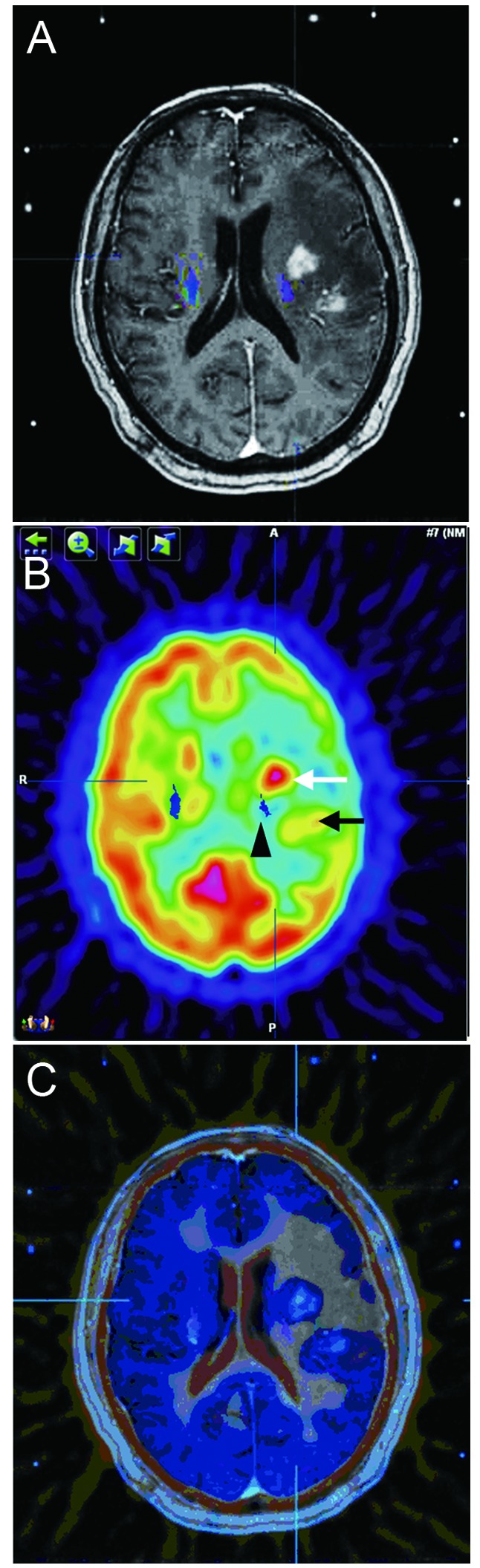
Sagittal fusion images. (A) Sagittal magnetic resonance imaging (MRI) obtained following gadolinium administration revealing two abnormal contrast-enhanced lesions located in the left basal ganglia areas. (B) Sagittal ^18^F-fluorodeoxyglucose (FDG)-positron emission tomography (PET) fusion image revealing higher FDG uptake within the upper right lesion (white arrow) than in the lower left lesion (black arrow) and the compressed left corticospinal tract of the posterior limb of the internal capsule (black arrowhead). (C) Sagittal PET-MRI fusion image demonstrating the overlap of the areas of high FDG uptake and the contrast-enhanced lesion.

**Figure 2:**
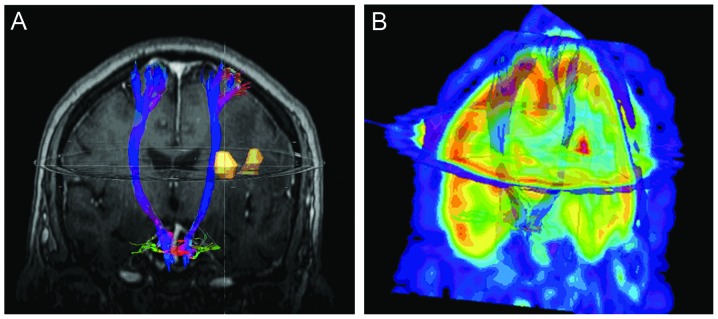
BrainLAB planning software was used to reconstruct the corticospinal tracts (CST) and reveal the three-dimensional (3D) anatomical association between the CST and the brain lesions, according to the ^18^F-fluorodeoxyglucose-positron emission tomography (PET) and contrast-enhanced magnetic resonance imaging (MRI) fusion images. (A) MRI-diffusion tensor imaging (DTI) fusion image revealing the 3D association between the lesion and the CST. (B) PET-DTI fusion image revealing the 3D association between the lesion and the CST.

An accurate could not be made based upon clinical presentation and imaging studies alone. Therefore, a high-field iMRI-guided stereotactic brain biopsy was performed five days after admission. The pre-operative and intraoperative contrast-enhanced MRI and DTI data were acquired with a 1.5T high-field iMRI scanner (Signa HDi; GE Healthcare, Pittsburgh, PA, USA). The high-field iMRI data collection, surgical planning and stereotactic brain biopsy were performed on the same day. The procedure was performed under local anesthetic using a Leksell G coordinate frame (Elekta, Stockholm, Sweden). The patient was placed on an MRI-compatible operating table in the MRI scanner room, which was transferred between the MRI scanner room and the surgical room through an MRI screening door. Following the scan, the digital imaging data and pre-operative PET/CT data were transferred to the navigation planning workstation software, iPlan 3.0 (BrainLAB, Feldkirchen, Germany), for automatic multi-image fusion (Fig. 1A–C). Subsequent to confirmation of the accuracy of the multi-image fusion, the navigation planning workstation software was used to reconstruct the corticospinal tracts (CSTs) for fibre tracking, and to define the lesion range according to the FDG-PET and contrast-enhanced MRI fusion images. The software was then used to generate the three-dimensional (3D) objects, which displayed the anatomical association between the CST and the brain lesions (Fig. 2A and B).

**Figure 3:**
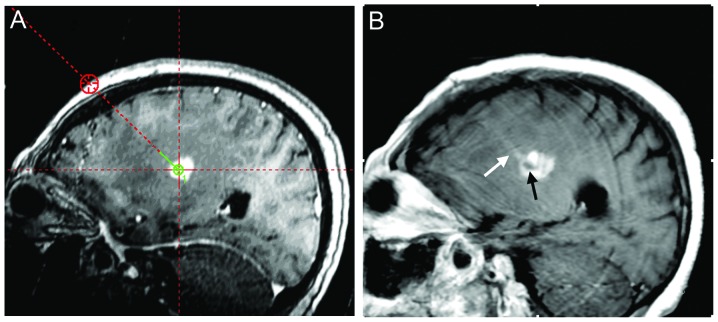
Comparision of pre-operative and intraoperative contrast-enhanced magnetic resonance imaging (MRI) images. (A) The ideal target site and a reasonable needle track were identified using the Leksell stereotaxic planning system. (B) The intraoperative MRI (iMRI) image revealing the biopsy trajectory (white arrow). Immediate iMRI identified no surgical bleeding, and the biopsy trajectory reached the target site (black arrow).

In addition, the contrast-enhanced MRI and pre-operative PET/CT data were transferred into the stereotaxic planning system software, Leksell SurgiPlan 10.1 (Elekta), for image fusion. Using the Leksell stereotaxic planning system, the ideal target site, a reasonable needle track (Fig. 3A) and the final target and trajectory coordinates of the stereotactic FDG-PET and contrast-enhanced MRI fusion images were identified. Simultaneously, the patient was transferred into the surgical room where a burr-hole was drilled and the stereotaxic biopsy was performed using a Sedan side-cutting biopsy needle (Elekta).

The 1.5T iMRI was used to effectively monitor the intracranial condition during the brain biopsy procedure. The immediate post-operative MRI identified no surgical bleeding or other complications, and comparison of the pre-operative planning imaging was performed to ensure that the target biopsy trajectory had been achieved (Fig. 3B). Upon post-operative examination, the original symptoms of the patient were not aggravated and no new neurological deficits were apparent. Histopathological examination of the biopsy specimen revealed sheets of large diffuse lymphoblastic cells with ovoid vesicular nuclei and prominent nucleoli with indistinct cell borders. In addition, immunohistochemistry revealed large neoplastic cells which were positive for CD19, CD20 and B-cell lymphoma 2. Therefore, the histopathological diagnosis of B-cell non-Hodgkin’s lymphoma was made. The patient received systemic chemotherapy: cyclophosphamide 800 mg/m^2^ intravenously (IV) on days 1 and 2, doxorubicin 50 mg/m^2^ IV on day 1, vincristine 1.4 mg/m^2^ IV on days 1 and 8, methotrexate 6720 mg/m^2^ IV on day 10. At the one-year follow-up, the patient was alive and free of disease.

## Discussion

Stereotactic biopsy, which relies on pre-operative imaging, is recognized as a safe and effective procedure (3,5,6). Due to accurate target localization and reliable immobilization, the frame-based stereotactic biopsy is accepted as a gold-standard technique (4,5). However, limitations of this approach exist; most notably the shifts in the integrity of intracranial structures. Brain shift may occur subsequent to the opening of the dura, biopsy needle insertion or cerebrospinal fluid loss, and can cause the target lesion to deviate away from the default planning target (3,7). In addition to the risk of brain shift, brain stereotactic surgery is rarely performed as open surgery. Therefore, in the present study, samples could not be taken under direct vision. As a result, the surrounding functionally-active normal tissue and cerebral vessels may be damaged, which could lead to intracranial bleeding and neurological deficits. These factors, compounded by lesion heterogeneity (8,9), reduce the positive biopsy rate, despite high-quality pre-operative imaging and the accurate frame-based stereotactic system.

During surgery, iMRI guidance for frame-based stereotactic brain biopsies has the ability to effectively solve the aforementioned issues, as the technique provides near real-time intraoperative imaging and allows surgeons to visualize the biopsy target and puncture track. iMRI scanning is performed according to the requirements of the surgeon and image data can be promptly updated in order to make comparisons with pre-operative images. In the event that the stereotactic biopsy track is not consistent with the pre-operatively planned track, surgeons may alter the surgical planning, adjust the puncture direction and take samples. Furthermore, the global status of the brain can be monitored in case of intraoperative complications, including intracranial hemorrhage and cerebral edema (10). In the present study, whilst the biopsy samples were being examined by the pathologist to confirm the presence of the diagnostic tissue, T2WI and turbo fluid-attenuated inversion recovery scans were performed to exclude the presence of biopsy-induced intraoperative bleeding.

The high-field iMRI system has advantages over the low-field system in that it provides more precise image data concerning the anatomical status of the neurovascular tissues. In addition, high-field iMRI affords advanced functional capabilities during the course of surgery, such as magnetic resonance angiography, venography and stimulation, diffusion-weighted and diffusion-tensor imaging (DTI), and perfusion and functional MRI (fMRI) (11,12). DTI is a novel fMRI technique that uses the diffusion energy (Brownian motion) of water molecules to map white matter tracts within the brain (13). Corresponding imaging software enables the white matter tracts to be visualized in 3D. The intraoperative visualization of white matter tracts has enabled the safe and effective resection of tumors in close proximity to the functional areas such as the eloquent cortex or optic radiation (14–16). In addition, DTI has proven reliable for the prediction of the clinical outcomes of patients with intracerebral hemorrhage (17). To the best of our knowledge, the present study was the first to apply DTI to the iMRI-guided stereotactic biopsy of basal ganglia region lesions. The DTI scan was used to trace the integrity of the subcortical white matter, which in this case was the CST, in order to avoid secondary damage during tissue extraction. The deep brain lesions were located in the area of the left basal ganglia and the left CST of the posterior limb of the internal capsule was compressed significantly (Fig. 1B). This principally resulted in a weakness of the patient’s right extremities. Therefore, a Sedan side-cutting biopsy needle was inserted into the center of the overlap between the high-FDG uptake and contrast-enhanced lesion areas. The lower-right tissue was avoided in order to prevent secondary damage to the CST (Fig. 1C). Following the stereotactic brain biopsy, the CST was preserved in post-operative DTI. This may explain why the original neurological symptoms of the patient were not aggravated and why further neurological dysfunction did not develop.

Using high-field iMRI, PET and MRI fusion images, guided stereotactic brain biopsies achieve an accurate histological diagnosis and avoid complications. DTI is an effective technique for the visualization of the CSTs. Therefore, this particular imaging technique may have a significant role in frame-based stereotactic biopsies of basal ganglia region lesions.
